# Resilient hepatic mitochondrial function and lack of iNOS dependence in diet-induced insulin resistance

**DOI:** 10.1371/journal.pone.0211733

**Published:** 2019-02-04

**Authors:** Pamela A. Kakimoto, Bruno Chausse, Camille C. Caldeira da Silva, José Donato Júnior, Alicia J. Kowaltowski

**Affiliations:** 1 Departamento de Bioquímica, Instituto de Química, Universidade de São Paulo, São Paulo, Brazil; 2 Departamento de Fisiologia e Biofísica, Instituto de Ciência Biomédicas, Universidade de São Paulo, São Paulo, Brazil; State University of Rio de Janeiro, BRAZIL

## Abstract

Obesity-derived inflammation and metabolic dysfunction has been related to the activity of the inducible nitric oxide synthase (iNOS). To understand the interrelation between metabolism, obesity and NO^**.**^, we evaluated the effects of obesity-induced NO^**.**^ signaling on liver mitochondrial function. We used mouse strains containing mitochondrial nicotinamide transhydrogenase activity, while prior studies involved a spontaneous mutant of this enzyme, and are, therefore, more prone to oxidative imbalance. Wild-type and iNOS knockout mice were fed a high fat diet for 2, 4 or 8 weeks. iNOS knockout did not protect against diet-induced metabolic changes. However, the diet decreased fatty-acid oxidation capacity in liver mitochondria at 4 weeks in both wild-type and knockout groups; this was recovered at 8 weeks. Interestingly, other mitochondrial functional parameters were unchanged, despite significant modifications in insulin resistance in wild type and iNOS knockout animals. Overall, we found two surprising features of obesity-induced metabolic dysfunction: (i) iNOS does not have an essential role in obesity-induced insulin resistance under all experimental conditions and (ii) liver mitochondria are resilient to functional changes in obesity-induced metabolic dysfunction.

## Introduction

Nitric oxide (NO^**.**^) is a gaseous membrane-permeable free radical that acts as a cellular signaling molecule through many mechanisms including activating soluble guanylyl cyclases, covalent modification of amino acids residues and lipids, scavenging of superoxide (forming peroxynitrite), and competing with molecular oxygen within mitochondrial Complex IV [1,2]. NO^**.**^ is synthesized mainly by nitric oxide synthase (NOS) family enzymes, which includes three isoforms that catalyze the reaction of arginine, NADPH and O_2_ to citrulline, NADP^+^ and NO^**.**^ [3]. NOS2 is the inducible nitric oxide synthase (iNOS) isoform, expressed under pro-inflammatory stimuli that activate the transcriptional factor NF-κB [4]. Conversely, calcium-dependent NOS1 and NOS3 are constitutively expressed. Upon induction of expression, iNOS has a much higher NO^**.**^ output than other NOSs, and is not controlled by Ca^2+^ [3,5].

Because of its high output and inducible characteristic, iNOS has been suggested to participate in inflammatory mechanisms associated with obesity [6], acting both within the physiopathology of the disorder and in the development of comorbidities [5,7]. In obese mouse livers, iNOS is found in hepatocytes as well as in macrophages/Kupffer cells [8]. Interestingly, insulin resistance induced by high fat diets (HFD) has been shown to be prevented by iNOS KO in mice [9], while its overexpression promotes liver steatosis and insulin resistance [10]. In a lipid infusion model, Charbonneau et al. demonstrated that fatty acids acutely promoted liver insulin resistance, increased hepatic glucose production and the nitration of important insulin downstream effectors (e.g. IRS1, IRS2 and AKT). All effects were prevented by iNOS KO [11]. Indeed, nitration and nitros(yl)ation of amino acids residues are important post-translational modifications that modulate metabolic pathways such as insulin signaling [12,13]. HFDs were shown to increase nitrotyrosine content in the liver [14], while a S-nitrosocysteine proteome analysis identified metabolic enzymes that are be S-nitros(yl)ated. The very long chain acyl-CoA dehydrogenase (VLCAD), an important β-oxidation enzyme, is one of the enzymes that can be S-nitros(yl)ated and, surprisingly, is activated by this modification at Cys238 [15]. Overall, these results consist of a strong set of evidence indicating that NO^**.**^ has significant roles in metabolic control resulting from HFDs.

NO^**.**^ may act in metabolic diseases by affecting mitochondria, central hubs for both the regulation of metabolism and oxidant production. As reviewed by Shiva et al., 2017, mitochondria and NO^**.**^ can interact at many different levels, since nitric oxide permeates membranes and may react directly with electron transport chain complexes, matrix enzymes, and superoxide radicals [16]. As such, disease-related NO^**.**^ and nutrient oversupply may compromise mitochondrial metabolic function.

In a recent review covering the effects of HFDs on liver mitochondria, we found that (i) many studies show prominent oxidative imbalance, (ii) some studies find NADH-linked or succinate-supported respiration to be decreased (while others do not), (iii) oxygen consumption was more susceptible to NO^**.**^ inhibition in one study [14], and (iv) some studies also find changes in fatty acid oxidation (reviewed in [17]). The discrepancies in prior studies are probably due to different protocols including in dietary composition and time on the HFD.

Notably, most HFD protocols do not compare their animals to those reared on synthetic diets with controlled content, using instead grain-based diets which may have variable nutritional composition, and differ from HFD in more than just the fat content [16]. It is also important to note that most studies in mice were conducted using the C57BL/6J mouse from Jackson Laboratories, a strain with a spontaneous mutation in the mitochondrial nicotinamide nucleotide transferase (NNT), which hampers the production of mitochondrial NADPH and thus impacts significantly on mitochondrial redox state [18]. This strain has been shown to be prone to the development of metabolic diseases [18–21].

Here, we aimed to evaluate the time-dependent roles of obesity-derived NO^**.**^ on hepatic mitochondrial function, by monitoring the effect of deletion of the iNOS enzyme in C57BL/6 mice, which contain normal NNT activity. Surprisingly, after 2, 4 and 8 weeks of high fat feeding to induce obesity, we did not observe any protection against loss of insulin sensitivity by iNOS absence, nor did we observe an overt, sustained, decline in liver mitochondrial function. We believe these findings, while negative and unexpected, are important to re-evaluate the suggested protagonist role of liver mitochondrial dysfunction and inflammatory NO^**.**^ signaling in the development of diet-induced insulin resistance.

## Materials and methods

### 1. Animal use and ethics

Experiments were conducted in agreement with the NIH Guidelines for the humane treatment of animals and were approved by the local Animal Care and Use Committee (*Comissão de Ética em Cuidado e Uso Animal*, *Instituto de Química*, *USP*; Permit number: 17/2013). Male, C57BL/6 wild type mice (WT, N = 64) and C57BL/6 iNOS knockout mice (KO, N = 65) were obtained from the *Biotério do Conjunto das Químicas* (University of São Paulo/SP) and genotyped by PCR for both iNOS KO and NNT presence (not shown). Animals were kept in air-filtered cages containing 3–4 animals with 12-hours light/dark cycles. Food and water were given *ad libitum*.

### 2. Diets

Known-component low and high fat diets (LFD and HFD, respectively) were manufactured by Pragsoluções Biociências (Brazil) based on AIN 93-M diets with some modifications [22–24], as shown in Table 1. At 8 weeks of age, regular chow was replaced by the LFD. After 2 weeks of acclimation, half of the animals were randomly kept on the LFD and half started the HFD. Body mass and food intake were monitored weekly. Food intake was converted to energy content as described in Table 1. Energy efficiency was estimated per animal as (mean body mass gain)/(food intake). After 2, 4 and 8 weeks on the HFD, mice were euthanized by cervical dislocation after overnight fasting and used for the experiments.

**Table 1:** Diet composition (adapted from [18–20]).

	Low Fat Diet (LFD)		High Fat Diet (HFD)	
	g/kg	g/1000 kcal	g/kg	g/1000 kcal
**Casein**	147.5	38.8	204.3	38.7
**L-Cistin**	1.9	0.5	2.6	0.5
**Corn starch**	435.1	114.6	0.0	0.0
**Dextrinized corn starch**	170.1	44.8	138.9	26.3
**Sucrose**	93.6	24.7	130.4	24.7
**Cellulose**	57.5	15.1	79.7	15.1
**Soy oil**	4.5	1.2	37.5	7.1
**Lard**	40.4	10.6	337.6	63.9
**Mineral mix**	36.9	9.7	51.5	9.8
**Vitamin mix**	10.5	2.8	14.7	2.8
**Choline bitartrate**	2.0	0.5	2.8	0.5
**BHT**	0.008	0.002	0.009	0.002
**Calculated energy content**	(kcal/kg)	3796.7		5280.7
**Protein**	(% Energy)	15.7		15.8
**Carbohydrates**		73.6		20.4
**Fat**		10.6		63.9

### 3. Indirect calorimetry and spontaneous physical activity

Eight-week LFD and HFD-fed mice were individualized in Columbus Instruments Comprehensive Lab Animal Monitoring System (CLAMS) cages for 24 hours for acclimation plus 24 hours for measurements. Oxygen consumption, CO_2_ production and locomotion were recorded for each animal every 4 minutes. Respiratory exchange ratios (RER) and energy expenditure (or “Heat”) were acquired by the equipment, respectively, as VCO_2_/VO_2_ and (3.815+1.232*RER)*VO_2_, as described in the manufacturer’s manual. Spontaneous physical activity (SPA) was calculated as the sum of the total XYZ axis infrared beam breaks.

### 4. Body composition

Eight-week LFD and HFD-fed mouse body composition was assessed by nuclear magnetic resonance in a Bruker's Minispec LF50 Body Composition Analyzer (WT N = 12, KO N = 9).

### 5. Insulin tolerance test

After 6 hours of morning fasting, insulin (0.6 units/kg body mass) was injected into the peritoneal cavity. Glucose concentrations were determined at 0, 10, 15, 30 and 60 minutes after insulin injection using a digital glucose meter (Roche). The analysis involved integrating the values obtained as a function of time. Measurements were done a few days prior to tissue isolations from the same animals, hence the slightly earlier time points.

### 6. Glucose tolerance test

After 6 hours of morning fasting, glucose (1 g/kg body mass) was administrated by gavage. Glucose concentrations were determined at 0, 15, 30, 60, 90 and 120 minutes after gavage using a digital glucose meter (Roche). The analysis involved integrating the values obtained as a function of time. Measurements were done a few days after ITT.

### 7. Mitochondrial isolation

Livers were finely minced immediately after dissection, washed in isolation buffer (250 mM Sucrose, 10 mM Hepes, 1 mM EGTA, pH 7.2) at 4°C and homogenized in a tissue grinder. The suspension was centrifuged at 800 g for 5 minutes and the resulting supernatant centrifuged at 12000 g for 10 min. The pellet was washed and resuspended in minimal volume of isolation buffer [25]. Outer mitochondrial membrane integrity was evaluated by quantifying cytochrome c using SDS-PAGE followed by Western Blotting (S1 Fig) and was equal in all samples.

### 8. Oxygen consumption

Freshly isolated mitochondria were incubated in respiratory buffer containing 120 mM sucrose, 65 mM KCl, 2 mM MgCl_2_, 1 mM KH_2_PO_4_, 1 mM EGTA, 10 mM Hepes, 0.1% fatty acid free BSA, pH 7.4, at 37°C, in an Oroboros O2K High Resolution Respirometer (Innsbruck, Austria). Oxygen consumption was monitored by sequential additions of ADP (1 mM) and the ATP synthase inhibitor oligomycin (1 μg.mL^-1^) to mitochondrial suspensions energized by respiratory substrates malate (2 mM), glutamate (2 mM) and succinate (2 mM) or malate (2 mM) and palmitoyl carnitine (12.5 μM). Oxygen consumption was normalized to total mitochondrial protein content (125 μg.mL^-1^) [25].

### 9. Complex I, II and glutamate dehydrogenase activities

Complex I and II activities were assessed in liver isolated mitochondria as described in Spinazzi et al., 2012, [26] with some modifications. Briefly, for complex I, 10 μg of mitochondrial protein were incubated in phosphate buffer (50 mM, pH 7.5) containing fatty acid free bovine serum albumin (3 mg.mL^-1^), antimycin A (1 μg.mL^-1^) and NADH (10 mM). The activity was monitored as the decrease in NADH absorbance after ubiquinone (10 mM) addition at 340 nm (ε = 6.2 mmol^-1^.cm^-1^). Complex II activity was obtained by incubating 4 μg of mitochondrial protein in phosphate buffer (25 mM, pH 7.5) containing fatty acid free bovine serum albumin (3 mg.mL^-1^), antimycin A (1 μg.mL^-1^), succinate (20 μM) and 2,6-dichlorophenolindolphenol (80 μM). The activity was followed as the decrease in 2,6-dichlorophenolindolphenol absorbance after decylubiquinone (50 μM) addition at 600 nm (ε = 19.1 mmol^-1^.cm^-1^). Glutamate dehydrogenase activity was assessed by incubating 10 μg of mitochondrial protein in Tris-HCl buffer (100 mM, pH 7.5) containing NH_4_Cl (50 mM) and NADH (0.2 mM). The activity was monitored as the decrease in NADH absorbance after α-ketoglutarate (2.5 mM) addition, as described in Herrero-Yraola et al., 2001 [27].

### 10. SDS-PAGEs and western blots

Isolated liver mitochondria were diluted in Laemli buffer and proteins were separated using a 10% polyacrylamide denaturating gel. Proteins were transferred to nitrocellulose membranes and incubated with 1:2000 anti-VLCAD, 1:1000 anti-CPT1, or 1:1000 anti-Cyt C. Anti-COX IV (1:1000) or Ponceau S staining were used as a loading controls. Fluorescent secondary antibodies (IRDye anti-mouse and anti-Rabbit, 1:20000) were added to the membranes and bands were obtained using a near-infrared Odissey System. Bands were semi-quantified using ImageJ densitometric analysis.

### 11. Serum measurements

Blood was harvested, kept room temperature for 30 minutes, and centrifuged 20 minutes for serum separation. Commercial kits were used, following the manufacturer’s instructions. Triglycerides and cholesterol were measured using LabTest (Brazil) kits. The ketone bodies kit was from Sigma-Merck, and the non-esterified fatty acid kit was from Wako Chemicals.

### 12. Materials

All reagents were obtained from Sigma-Merck, except for oligomycin (cat. sc-201551), CCCP (cat. sc-202984) and palmitoyl-L-carnitine (cat. sc-203176), from Santa Cruz, and insulin (Humulin R), from Eli Lilly. VLCAD (cat. ab155138), CPT-1 (cat. ab128568) antibodies from Abcam; COX IV (cat. 4844) antibodies from Cell Signaling: cytochrome c (cat. 612504) from Biolegend, and anti-mouse (cat. 926–68070) and anti-rabbit IRDye (cat. 926–68071) were from LI-COR Biotechnology. Triglycerides (cat. 87) and cholesterol (cat. 76) kits were from Labtest. The NEFA kit (cat. 434.71795) was from Wako Chemicals.

### 13. Statistics

Data are shown as means + SEM. Outliers were identified by the ROUT method (Q = 1%). Two-way analysis of variance (ANOVA), and Sidak’s post-test were used to compare differences between means. Differences were considered significant when p < 0.05. Repetitions (N) are indicated in the methods section.

## Results

### 1. iNOS KO mice present lower spontaneous activity and are more susceptible to body mass gain induced by HFD

**Fig 1:**
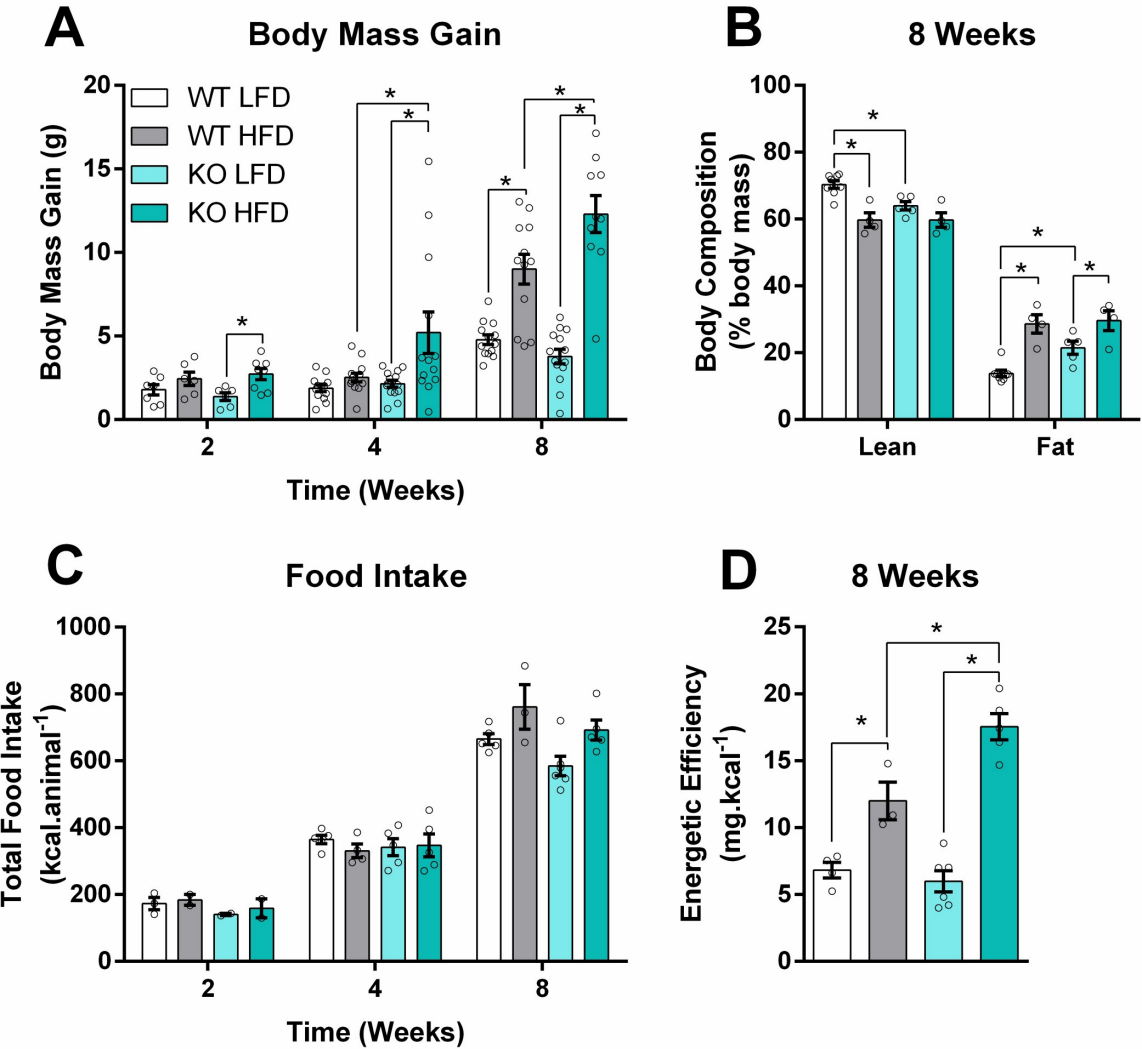
iNOS KO mice are more susceptible to body mass gain under HFDs. Body mass and food intake were monitored weekly. (A) Body mass gain 2, 4 or 8 weeks after the HFD started (n = 6–14). (B) Body composition assessed by nuclear magnetic resonance after 8 weeks of high fat feeding (n = 4–8). (C) Total food intake (n = 2–6 cages containing 3–4 mice) estimated as described in Material and Methods. (D) Energy conversion efficiency calculated as the ratio of body mass gain (in A) to food intake (in C) after 8 weeks. Data are mean + SEM. Unfilled circles represent biological replicates. Differences among means were evaluated by two-way ANOVA. * = p < 0.05 in Sidak’s posttest analysis.

We promoted obesity in mice using a HFD composed of 64% energy from fat, mainly derived from pork lard (90%, Table 1) and a corresponding known-component low fat diet (LFD). The HFD promoted body mass gains at all feeding periods tested in iNOS KO animals (Fig 1A), while in WT animals the gain was only significant after 8 weeks. The HFD also increased body fat mass in both groups (Fig 1B). Curiously, iNOS KO mice tended to eat less (genotype effect, P value = 0.041, Figs 1C and S2). The higher body mass gains they achieved combined with lower food intake indicate a high energy efficiency of iNOS KO mice compared to WT when fed the HFD, but not on the LFD (Fig 1D).

**Fig 2:**
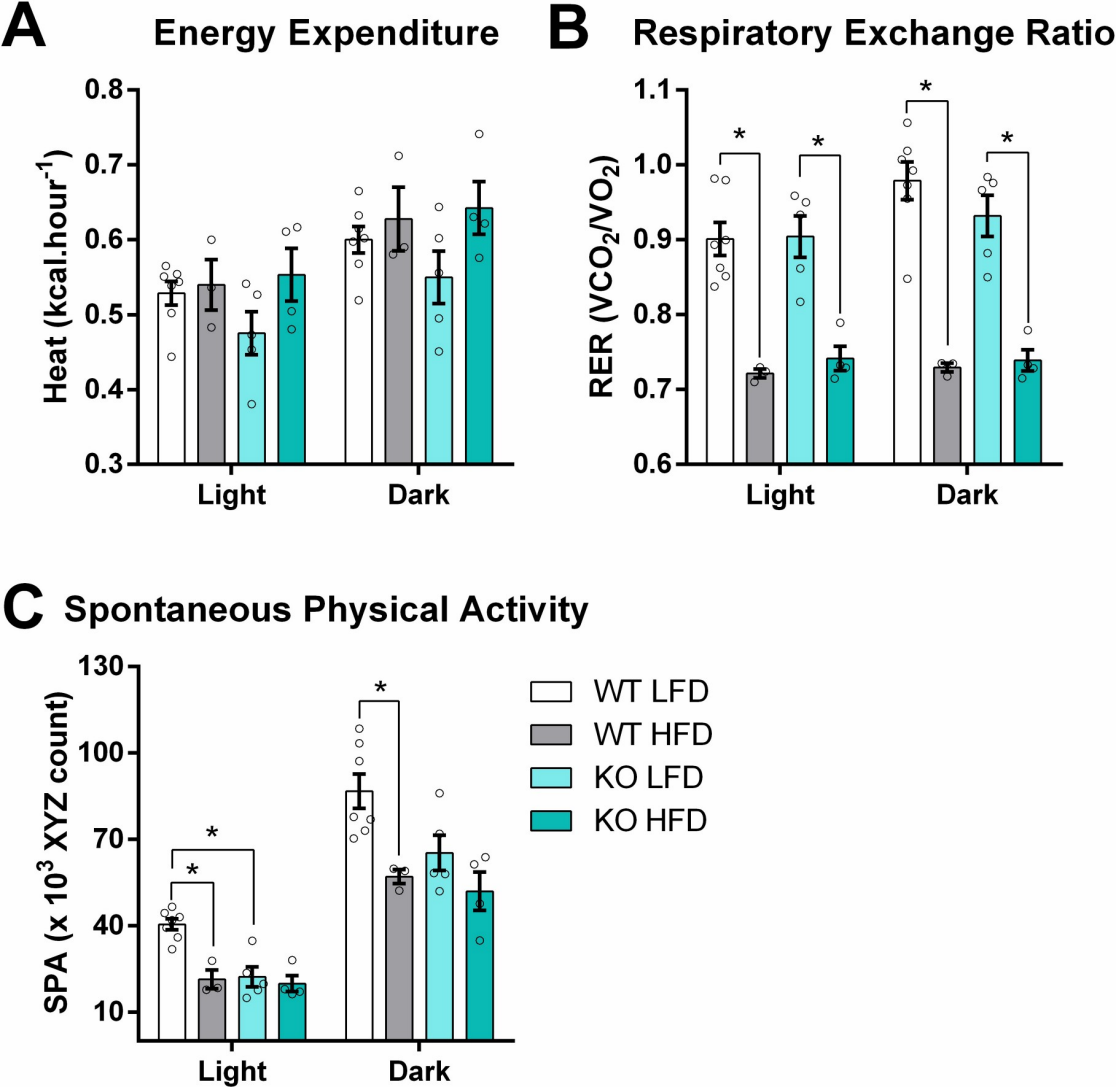
HFDs increase energy expenditure while decreasing spontaneous activity. Indirect calorimetry and SPA measurements were performed after 8 weeks of HFD feeding. Light and dark cycles represent means of uninterrupted 12 h periods (7 AM—7 PM and 7 PM– 7 AM, respectively). Heat (A) and RER (B) were quantified automatically while SPA (spontaneous physical activity; C) represents XYZ axes infrared beams breaks, as described in Material and Methods. Data are mean + SEM, n = 3–8. Unfilled circles represent biological replicates. Differences among means were evaluated by two-way ANOVA. * = p < 0.05 in Sidak’s posttest analysis.

Interestingly, KO mice showed the same energy expenditure compared to WT counterparts (Fig 2A). However, HFD-feeding promoted a shift in fuel utilization, increasing the use of fat, as indicated by lower respiratory exchange ratios (RER, Fig 2B), while reducing spontaneous physical activity in WT animals (SPA, Fig 2C). iNOS KO mice tended to move less during the light and dark periods (genotype effect, P value = 0.004 and 0.062, respectively), which corroborates the higher energy efficiency and mass gain observed.

### 2. iNOS absence protects from insulin resistance only on the LFD

**Fig 3:**
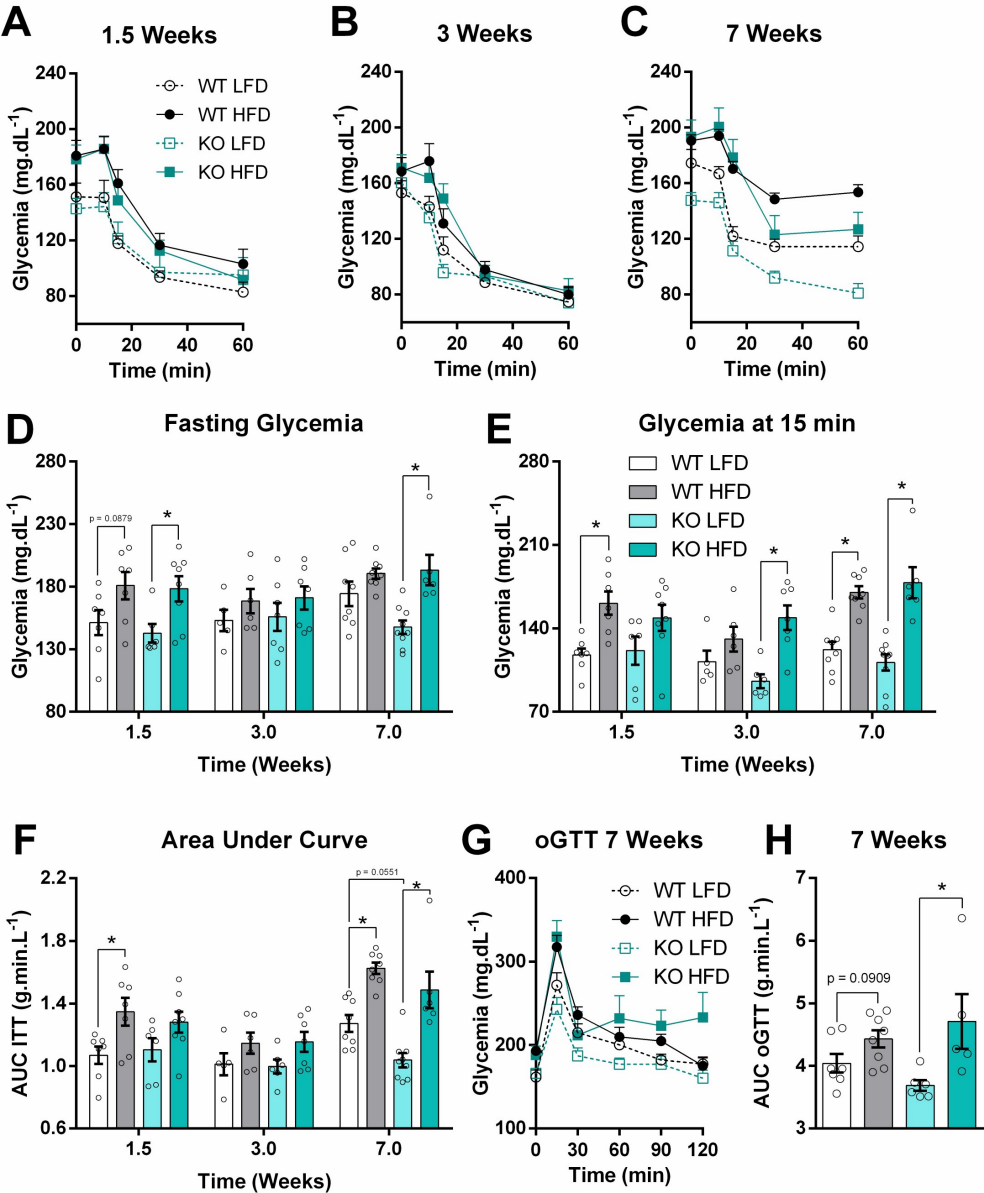
HF-feeding promotes insulin resistance despite iNOS absence. Insulin sensitivity was evaluated through insulin and glucose tolerance tests. (A-C) Time course glycemia after insulin injection in animals fed the HFD for 2, 4 or 8 weeks. (D) Glycemia after 6 hours of morning fasting. (E) Glycemia after 15 minutes of insulin injection. (F) Area under the curve for graphs A-C. (G) Time course glycemia after oral administration of glucose. (H) Area under the curve for graph F. Data are means + SEM, n = 5–9. Unfilled circles represent biological replicates. Differences among means were evaluated by two-way ANOVA. * = p < 0.05 in Sidak’s posttest analysis.

Systemic insulin sensitivity was evaluated using insulin and oral glucose tolerance tests at different time points (ITT and oGTT, respectively, Fig 3). As expected, the HFD decreased insulin sensitivity after 1.5 weeks in WT animals, as measured by the glycemic response at 15 min (Fig 3E) and the ITT area under the curve (AUC, Fig 3F). Fasting glycemia was higher in the iNOS KO HFD group at 1.5 weeks (Fig 3D), despite no significant changes in the ITT 15 min glycemia (Fig 3E) nor the ITT AUC (Fig 3F). Insulin sensitivity is significantly reduced in both WT and KO groups at 7 weeks, a time point in which the oGTT also showed changes promoted by the HFD (Fig 3G and 3H). Interestingly, even LFD WT animals tended to be less sensitive to insulin at 7 weeks, suggesting that diets with only 10% energy from fat can already promote metabolic disturbances when ingested *ad libitum*. These effects were not observed in LFD iNOS KO mice. Conversely, iNOS KO mice have increased circulation of NEFA and ketone bodies after overnight fasting, both on high- and low-fat diets (S1 Table)

### 3. Glutamate, succinate, and palmitoyl-L-carnitine-supported oxygen consumption are decreased by HFDs, but recover over time

**Fig 4:**
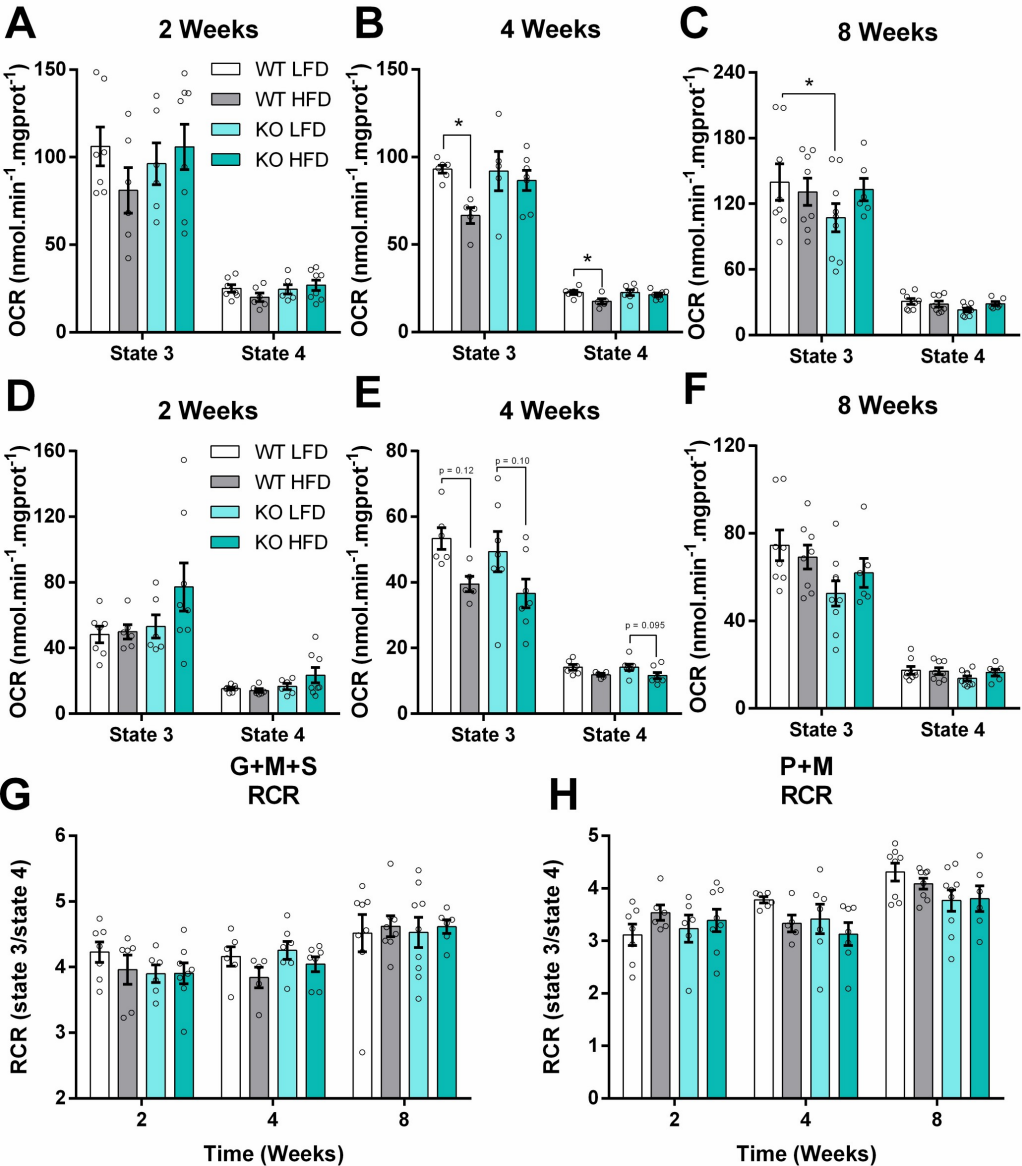
Glutamate, succinate, and palmitoyl-L-carnitine-supported oxygen consumption decreased mildly with HFD, but recovered over time. Oxygen consumption rates (OCR) of isolated mitochondria from livers after 2, 4 and 8 weeks of high-fat feeding. (A, B and G) Glutamate, malate and succinate were used as substrates. (D, F and H) Palmitoyl-carnitine and malate as substrates. “State 3” is the oxygen consumption rate in presence of ADP; “state 4” represents oxygen consumption measured in the presence of ATP synthase inhibitor oligomycin. Data are means + SEM, n = 5–9. Unfilled circles represent a biological replicate. Differences among means were evaluated by two-way ANOVA. * = p < 0.05 in Sidak’s posttest analysis.

Mitochondrial function was assessed by oxygen consumption measurements under different functional states (Fig 4). Liver mitochondria were isolated and energized by complex I and II substrates glutamate and succinate, respectively, and respiration was measured in the presence (state 3) and absence (state 4) of ATP production, promoted by the addition of ADP and ATP-synthase inhibitor oligomycin, respectively (S3 Fig). The HFD slightly decreased state 3 and 4 respiration at 4 weeks in WT mitochondria (Fig 4A versus Fig 4B), although this was recovered at 8 weeks (Fig 4C). No changes were promoted by the diets on respiratory control ratios (RCR, Fig 4G), a measure of mitochondrial oxidative phosphorylation efficiency. In line with these findings, no changes in Complex I, II, and glutamate dehydrogenase enzymatic activities were observed (Table 2).

**Table 2:** Complex I, II and glutamate dehydrogenase activities.

	WT LFD			WT HFD			KO LFD			KO HFD		
	Activity (μM.min^-1^.μg protein)											
	Mean	SEM	N	Mean	SEM	N	Mean	SEM	N	Mean	SEM	N
	2 Weeks											
**Complex I**	0.35	0.02	4	0.29	0.04	5	0.41	0.03	5	0.35	0.04	5
**Complex II**	1.83	0.26	6	1.86	0.28	6	2.00	0.18	6	1.94	0.15	6
**GDH**	1.14	0.06	5	1.18	0.08	5	1.24	0.03	5	1.28	0.04	5
	4 Weeks											
**Complex I**	0.35	0.02	5	0.32	0.01	5	0.32	0.03	5	0.31	0.02	5
**Complex II**	1.95	0.07	6	1.85	0.02	6	2.02	0.24	6	1.94	0.16	6
**GDH**	1.15	0.11	5	1.24	0.05	5	1.12	0.12	4	1.19	0.06	5
	8 Weeks											
**Complex I**	0.41	0.04	5	0.40	0.06	5	0.35	0.02	5	0.34	0.01	5
**Complex II**	1.93	0.03	6	1.87	0.11	6	1.88	0.11	6	1.87	0.08	6
**GDH**	1.11	0.07	5	1.31	0.05	5	1.23	0.17	4	1.36	0.11	4

Data are: Mean, Standard error of the mean (SEM), and the Number of biological replicated (N). The means at the same animal age are not statistically different (p < 0.05). Two-way ANOVA.

**Fig 5:**
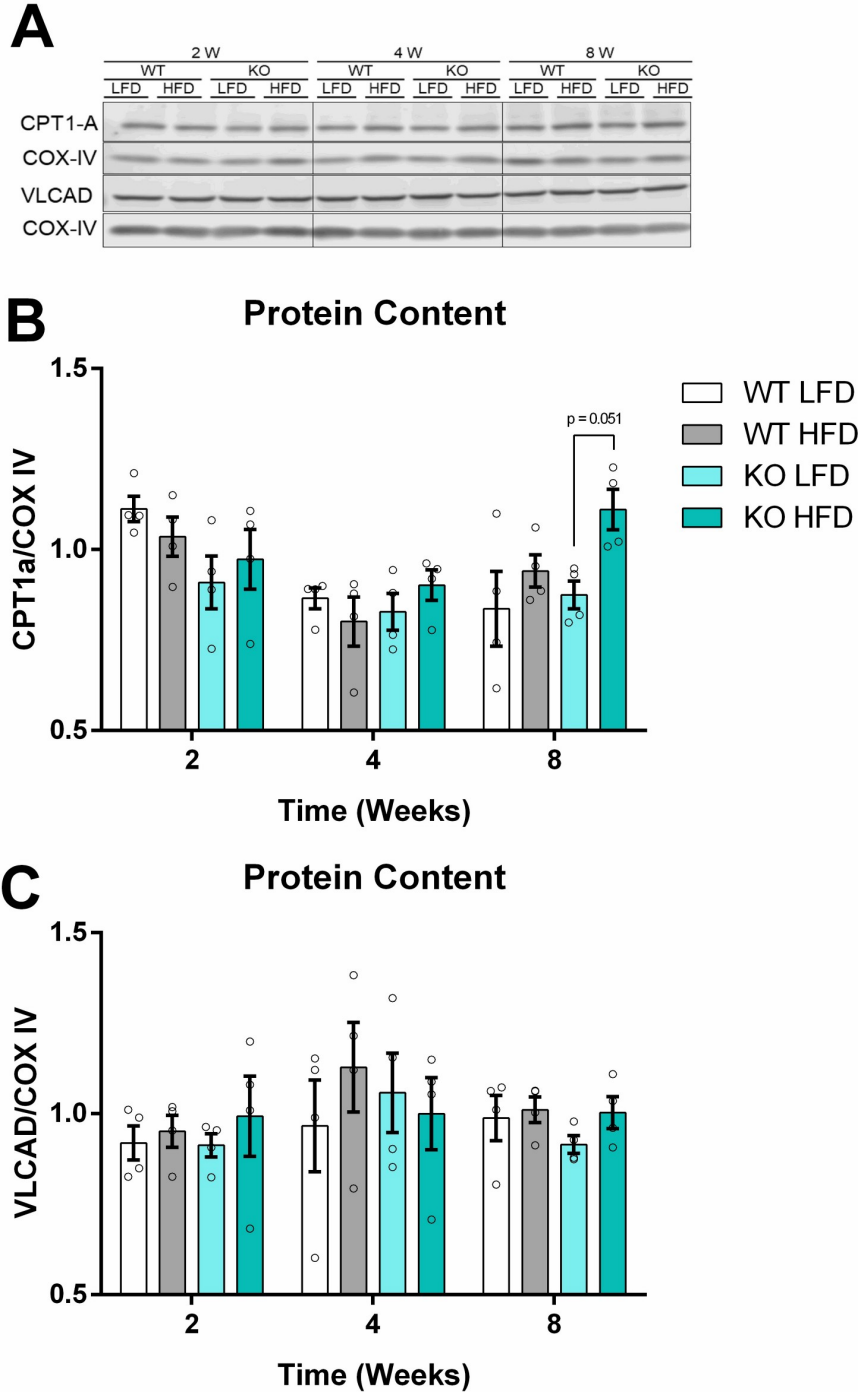
HFD increases CPT1-A content. SDS-PAGE Western blots of liver mitochondria from animals fed the HFD for 2, 4 or 8 weeks. Representative bands in (A) and quantification by densitometry in (B) and (C). Data are means + SEM, n = 4. Unfilled circles represent a biological replicate. Differences among means were evaluated by two-way ANOVA and Sidak’s posttest analysis.

A similar trend in lower respiration at 4, but not 8 weeks, was observed with respiration supported by palmitoyl-L-carnitine, a long chain fatty acid (LCFA) activated with L-carnitine that readily enters the mitochondrial matrix (Fig 4D–4F), albeit independently of iNOS absence. Again, no changes were promoted by the diets on fatty acid-supported respiratory control ratios (Fig 4H). Carnitine palmitoyl transferase 1A (CPT1-A) protein levels tended to be increased in the KO HFD group at 8 weeks of feeding (Fig 5A and 5B). No changes were observed in the protein levels of very long chain acyl-CoA dehydrogenase (VLCAD, Fig 5A and 5C), a result compatible with the lack of change in fatty acid-supported respiration.

## Discussion

High fat diets are a widely used model to induce obesity that mirrors, in rodents, many of the effects of human overnutrition and lack of physical activity. Indeed, we identified that after 1.5 weeks on a HFD, mice already exhibited increased body mass and adiposity. iNOS KO mice were more susceptible to body mass gain and had an increased content of fat mass even on the LFD (Fig 1). Interestingly, iNOS KO animals were found to be spontaneously hypercholesterolemic [28], a finding that we also observed at 4 weeks in both LFD and HFD groups (S1 Table). In addition, iNOS KO mice have increased circulation of NEFA and ketone bodies after overnight fasting (S1 Table). These findings corroborates with the already described susceptibility to increased fat mass [9] and higher energetic efficiency of these animals (Fig 1D), suggesting differential lipid handling in iNOS KO animals that may be exacerbated under HFDs.

Unexpectedly, iNOS KO did not protect from HFD-induced insulin resistance even at 1.5 weeks (Fig 3), although the deleterious effects of iNOS on insulin sensitivity have been widely described in mice [9–11,29,30]. Part of the discrepancies between our results and those from other laboratories may be due to at least two important differences when rodent obesity models are considered [31,32]: 1) *the genetic background*—our WT and KO animals are Nnt+/+ backgrounds while some of the other groups used the spontaneously mutated Nnt-/- mice from Jackson Laboratories [9,29]. The NNT (nicotinamide nucleotide translocase) enzyme promotes re-reduction of NADPH required for the removal of mitochondrial oxidants, and its absence may promote changes in redox signaling in the presence/absence of nitric oxide [20,33,34]; 2) *the formulation and timing of the HFD provided—*both diets (HFD and LFD) we used are composed mainly of lard and soybean oil (9:1) as lipid sources, and the HFD was able to reduce insulin sensitivity in only 1.5 weeks. Using a similar approach, Nozaki and colleagues observed protection of iNOS absence after 10 and 48 weeks of a HFD containing 57.5% of energy from fat derived from beef tallow and safflower oil (~3:4). Pork lard contains a higher proportion of monounsaturated fatty acids than saturated fatty acids, contrary to beef tallow, and different types of triacylglycerols [35]. Safflower oil can be rich in linoleic acid or in oleic acid, depending on the crop used for oil extraction [36,37], while soybean oil is consistently linoleic acid-enriched. The different HFD composition due to the source of fats and also the slightly higher content of sucrose (~10% in our diets versus ~5% in theirs) could explain the disparities seen, since some authors already described differences in insulin sensitivity and adipose tissue inflammation modulating just fatty acid types, but not total quantities [38–40]. Overall, the fact that iNOS is not necessary for metabolic responses under all conditions described to date suggests that its role as a protagonist in the pathology of obesity should be reconsidered.

Of note, we see signs of decreased insulin sensitivity in WT LFD animals after 7 weeks, an effect probably related to the fact that, despite being low in fat, the diet is offered *ad libitum* and animals are not exercised, resulting in mild obesity [38]. In contrast, iNOS KO LFD animals have lower spontaneous physical activity, while showing higher fat mass and insulin sensitivity. Thus, some of the beneficial effects of iNOS absence seen by other authors were in partially mirrored in our model using the LFD. A recent paper by Kanuri and co-authors observed, in the spontaneously-mutated NNT-deficient background, that iNOS KO animals on a LFD (10% energy from fat, mainly soybean oil) had reduced energy expenditure and RER compared to WT animals after 5 weeks of feeding [41]. They propose that there are optimal iNOS/eNOS/nNOS relations that maintain glucose and lipid homeostasis and that iNOS KO could compromise basal homeostatic levels of NO^**.**^, a hypothesis that finds resonance with our data.

One of the roles of the liver in type 2 diabetes is unregulated glucose production [42], contributing toward hyperglycemia. Some authors hypothesize that liver mitochondria have a central role coordinating fuel substrate use under these conditions [43–45]. We evaluated the relationship between systemic insulin resistance and liver mitochondrial function, and found a non-significant trend toward lower state 3 oxygen consumption with a fatty acid as substrate at 4 weeks (Fig 4E), which is parallel to a discrete decrease in the ITT area under the curve at 3 weeks on the HFD (Fig 3B). Shulman’s group proposed a mechanism in which hepatic glucose production is indirectly controlled by lipolysis in the white adipose tissue, increasing fatty acid flow to the liver and culminating in a surplus of acetyl-CoA through β-oxidation and subsequent allosteric activation of pyruvate carboxylase, the first step of hepatic gluconeogenesis [46]. In line with the idea that mitochondrial integrity is preserved, with enhanced β-oxidation activity, we observed that mitochondrial function is fully recovered and more coupled after 8 weeks on the HFD using complex I and II substrates, as well as fatty acids (Fig 4), while insulin sensitivity is even more compromised (Fig 3C). Similar findings were obtained by Franko and co-authors (2014) using three different models of insulin resistance, agreeing with the resilient function of liver mitochondria that we observed [47]. Additionally, although palmitoyl-L-carnitine oxidation may be independent of CPT1-mediated transport, the increased content of this protein (Fig 5A and 5B) suggests a higher lipid oxidation capacity *in situ*. Overall, literature data and our findings strongly support the idea that liver mitochondrial integrity is largely maintained in obesity-mediated insulin resistance, with enhanced lipid oxidation capacity but no functional damage.

In conclusion, we observed that our HFD can induce metabolic disturbances over a short period of time, while the LFD control is also not innocuous, decreasing insulin sensitivity at 7 weeks. We believe that this LFD effect should always be expected and considered in time-point assessments of metabolic effects of high fat diets, since animals on *ad libitum* feeding tend to be obese even with chow diets [48]. Furthermore, we replicated the tendency of iNOS KO animals to gain more body mass under HFDs, but failed to show any protection against insulin sensitivity, suggesting iNOS is not essential for insulin resistance development under all conditions. In parallel with systemic insulin resistance, we uncovered that hepatic mitochondrial function is sustained over time. We thus believe that the observation of mitochondrial dysfunction by some authors may be a consequence of systemic decline and not its cause. Together, these results demonstrate that neither iNOS nor hepatic mitochondrial damage are indispensable players in all forms of HFD-mediated insulin resistance.

## Supporting information

S1 Fig Mitochondrial outer membrane integrity. Cytochrome c content was evaluated by SDS-PAGE Western Blotting isolated liver mitochondria from 8-week old HF-fed animals. Densitometric semiquantitative analysis was performed in ImageJ. Data are mean + SEM, n = 4. Unfilled circles represent biological replicates. Differences among means were evaluated by two-way ANOVA and are not significant for p < 0.05.
(TIF)

S2 Fig Total food intake over time. Food intake was monitored weekly. Total food intake (N = 2–6 cages containing 3–4 mice) was estimated as described in Material and Methods. Data are mean + SEM. Unfilled circles represent biological replicates. Differences among means were evaluated by two-way ANOVA. * = p < 0.05 in Sidak’s posttest analysis.
(TIF)

S3 Fig Representative oxygen consumption assay. Isolated liver mitochondrial oxygen consumption after 4 weeks of high-fat feeding. (A) Glutamate, malate and succinate were used as substrates. (B) Palmitoyl-carnitine and malate were used as substrates. “State 3” is the oxygen consumption rate in presence of ADP; “state 4” represents oxygen consumption measured in the presence of ATP synthase inhibitor oligomycin.
(TIF)

S1 Table iNOS KO mice are hypertriglyceridemic and hypercholesterolemic. (DOCX)
