# Supplementary figures and images for: Resilient hepatic mitochondrial function and lack of iNOS dependence in diet-induced insulin resistance
Source: PLoS One. 2019 Feb 4;14(2):e0211733. doi: 10.1371/journal.pone.0211733 (PMC6361450; doi:10.1371/journal.pone.0211733)

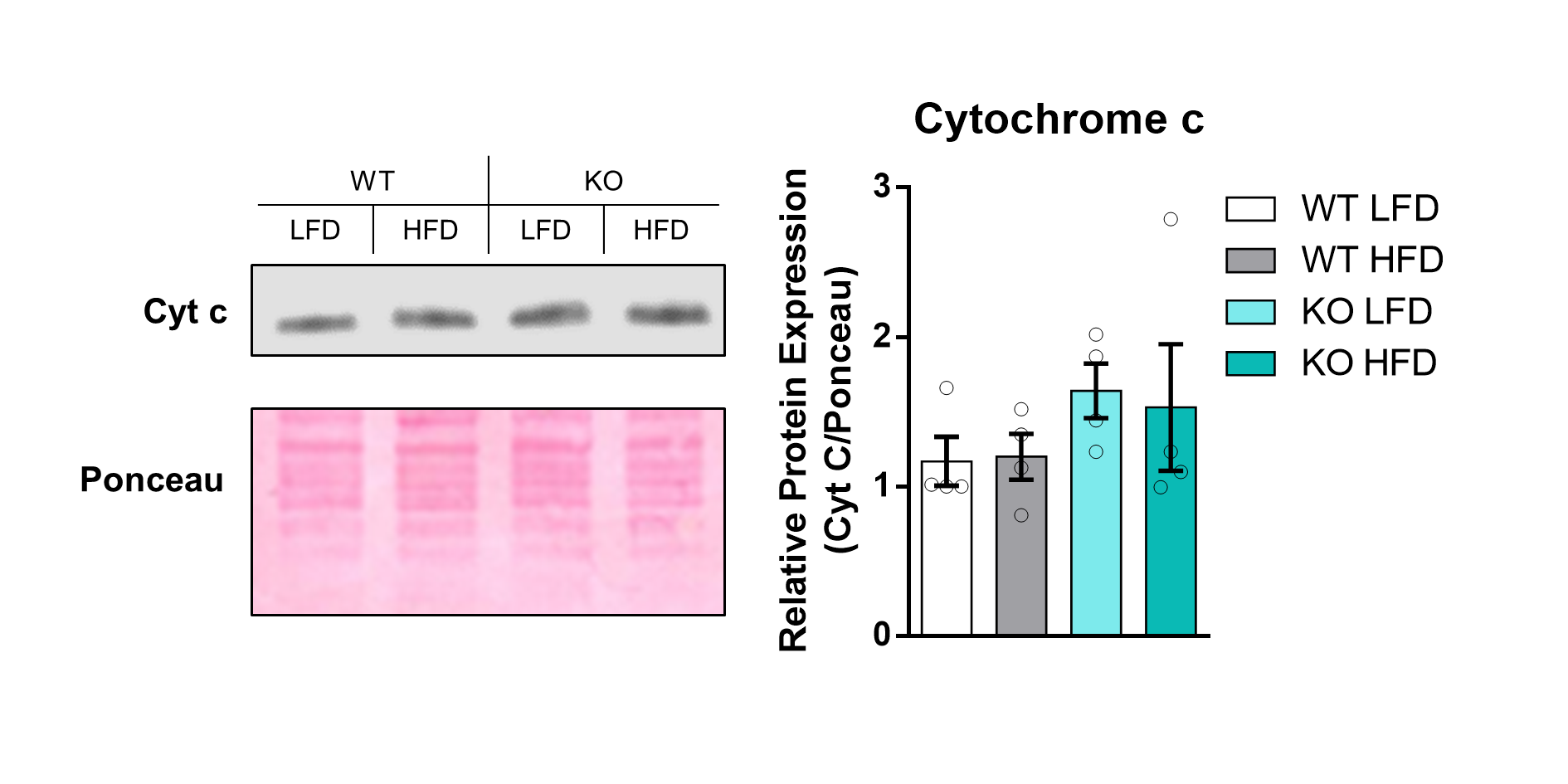

Supplement: S1 Fig — Cytochrome c content was evaluated by SDS-PAGE Western Blotting isolated liver mitochondria from 8-week old HF-fed animals. Densitometric semiquantitative analysis was performed in ImageJ. Data are mean + SEM, n = 4. Unfilled circles represent biological replicates. Differences among means were evaluated by two-way ANOVA and are not significant for p < 0.05. (TIF) [file pone.0211733.s001.tif]
