# Supplementary material for: Resilient hepatic mitochondrial function and lack of iNOS dependence in diet-induced insulin resistance
Source: PLoS One. 2019 Feb 4;14(2):e0211733. doi: 10.1371/journal.pone.0211733 (PMC6361450; doi:10.1371/journal.pone.0211733)
Supplement: S1 Table — (DOCX) [file pone.0211733.s004.docx]

**S1 Table:**

|  | WT LFD | | | WT HFD | | | KO LFD | | | KO HFD | |  |  |
| --- | --- | --- | --- | --- | --- | --- | --- | --- | --- | --- | --- | --- | --- |
|  | Mean | SEM | N | Mean | SEM | N | Mean | SEM | N | Mean | SEM | N | 2-way ANOVA |
| Triglycerides  (mg.dL^-1^) | 94.64 | 10.35 | 5 | 61.29 | 6.11 | 6 | 136.63 | 13.02 | 5 | 117.59 | 11.2 | 5 | *,# |
| Cholesterol  (mg.dL^-1^) | 94.77 | 5.87 | 5 | 84.94 | 4.68 | 6 | 117.07 | 4.74 | 5 | 119.46 | 5.37 | 5 | # |
| NEFA (µM) | 5.93 | 1.49 | 4 | 5.21 | 0.59 | 4 | 9.71 | 0.74 | 4 | 9.84 | 2.05 | 4 | # |
| Ketone Bodies (mM) | 1.08 | 0.18 | 6 | 0.87 | 0.23 | 6 | 1.61 | 0.17 | 6 | 1.20 | 0.13 | 6 | # |

Serum content after 4 weeks of HFD and overnight fasting.

Data are: Means, Standard error of the mean (SEM), and the Number of biological replicated (N). Two-way ANOVA, * = diet effect, # genotype effect, p < 0.05.
